# Quantitative Study of Morphological Features of Stem Cells onto Photopatterned Azopolymer Films

**DOI:** 10.3390/jfb11010008

**Published:** 2020-02-14

**Authors:** Marcella Salvatore, Stefano Luigi Oscurato, Marietta D’Albore, Vincenzo Guarino, Stefania Zeppetelli, Pasqualino Maddalena, Antonio Ambrosio, Luigi Ambrosio

**Affiliations:** 1 Physics Department “E. Pancini”, Università degli Studi di Napoli “Federico II”, Complesso Universitario di Monte Sant’Angelo, Via Cintia, 80126 Naples, Italy; marcella.salvatore@unina.it (M.S.); oscurato@fisica.unina.it (S.L.O.); pasmad@fisica.unina.it (P.M.); 2 Former Temporary Researcher at Institute of Composite and Biomedical Materials, National Research Council of Italy, Viale Marconi 4, 80125 Naples, Italy; 3 Institute of Polymers, Composites and Biomaterials, National Research Council of Italy, Mostra D’Oltremare, Pad.20, V.le J.F. Kennedy 54, 80125 Naples, Italy; stefania.zeppetelli@cnr.it (S.Z.); luigi.ambrosio@cnr.it (L.A.); 4 CNST@POLIMI—Fondazione Istituto Italiano di Tecnologia, Via Pascoli 70, 20133 Milano, Italy

**Keywords:** azopolymers, reconfigurable materials, cell-instructive materials, cell orientation

## Abstract

In the last decade, the use of photolithography for the fabrication of structured substrates with controlled morphological patterns that are able to interact with cells at micrometric and nanometric size scales is strongly growing. A promising simple and versatile microfabrication method is based on the physical mass transport induced by visible light in photosensitive azobenzene-containing polymers (or azopolymers). Such light-driven material transport produces a modulation of the surface of the azopolymer film, whose geometry is controlled by the intensity and the polarization distributions of the irradiated light. Herein, two anisotropic structured azopolymer films have been used as substrates to evaluate the effects of topological signals on the in vitro response of human mesenchymal stem cells (hMSCs). The light-induced substrate patterns consist of parallel microgrooves, which are produced in a spatially confined or over large-scale areas of the samples, respectively. The analysis of confocal optical images of the in vitro hMSC cells grown on the patterned films offered relevant information about cell morphology—i.e., nuclei deformation and actin filaments elongation—in relation to the geometry and the spatial extent of the structured area of substrates. The results, together with the possibility of simple, versatile, and cost-effective light-induced structuration of azopolymers, promise the successful use of these materials as anisotropic platforms to study the cell guidance mechanisms governing in vitro tissue formation.

## 1. Introduction

The extracellular matrix (ECM) enormously contributes to the ultimate properties of tissues and organs [1]. By a complex assembly of pores, ridges, and fibers at the nanometric scale, it can regulate several biological functions. Hence, a controlled patterning of the surfaces at sub-micrometer or nanometer scale is currently considered for the design of a new biomaterials generation—namely, cell instructive materials (CIMs) [2] with improved functionalities and bioactive properties to mimic the natural ECM of tissues. In the last years, several groups have reported about the relevance of substrate topography to address cell response, in terms of adhesion, spreading, and differentiation [3]. Hence, an increasing number of fabrication strategies is emerging to design functional materials that respond to a set of different stimuli (electrical, magnetic, topographic) in order to instruct cells for specific biological functions [4,5].

In this context, soft lithographic techniques such as micro-contact printing (µCP), replica molding (REM), micro-transfer molding (µTM), micro-molding in capillaries (MIMIC), and solvent-assisted micro-molding (SAMIM) have been commonly used for fabricating high-quality microstructures and nanostructures [6,7,8], offering the chance to achieve a wide variety of topographical features (i.e., grooves, pillars, gratings, tubes, pits, and spheres). With advances in chemistry and material science, the use of light-based fabrication technologies (i.e., photolithography [9]) in this field is drastically increasing [10]. However, despite the ability to precisely design even complex surface patterns and to reproduce them with high quality, standard photolithographic techniques are often expensive and time-consuming, usually requiring several and demanding steps of fabrication. A promising microfabrication method, which overcomes several limitations of the standard photolithographic approaches, is based on the light-induced mass transport promoted in azobenzene-containing polymers (or simply azopolymers) by irradiation of structured visible light [11]. Under light irradiation, the azobenzene molecules sustain cyclical trans-cis-trans isomerization reactions that force a macroscopic displacement of the polymer matrix in which they are embedded. The consequence of this molecular light-fueled material motion is the macroscopic structuration of azopolymer films and microvolumes, whose geometry depends on the intensity and polarization distributions of the irradiated light field [11,12,13,14,15]. Consequently, azopolymers can be directly structured over large scales with high quality, in spatially selective, simple, and cost-effective way, creating spatially ordered and even complex [16] topological patterns exploitable as microstructured platforms to control cellular orientation and migration in in vitro cultures [17,18]. Additionally, due to the non-destructive nature of the light-induced mass migration phenomenon, the structuration of the azopolymers is reversible [19]. This feature permits the peculiar possibility of repeatedly changing the topography of the surface through light irradiation, ideally allowing even the remotely real-time variation of topographic stimuli, which can be crucial for applications in biomedical fields [20]. Moreover, the use of the azobenzene-containing polymers as substrates for cell guidance and ECM mimicking can take advantage also of the reversible switch of their electric properties produced, simultaneously to the macroscopic mass displacement, by the photo-orientation of the azobenzene molecules under illumination [16,21]. This effect can indeed enhance the conjugation of polymeric chains [22,23,24], increasing the charge carrier mobility and the ionic conductivity in aqueous solutions.

Recent studies demonstrate that cell guidance—i.e., capability to adjust cell orientation and alignments along patterns [25]—can be explained by the orientation of focal contacts influencing the cell spreading [26,27] and the structural changes of cell nuclei [28]. This correlation is still partially unexplored, but it seems to be determined by stress–strain states in the cytoskeleton that are able to influence chemical (i.e., transcription of specific genes), or physical (i.e., permeability of nuclear membrane [29]) signals. Starting from this background, we provide here a qualitative/quantitative morphological study via image analysis aimed at exploring the contribution of anisotropic structured topography of an azopolymer film on the in vitro response of human mesenchymal stem cells (hMSCs).

## 2. Materials and Methods

### 2.1. Azopolymer and Film Preparation

**Figure 1:**
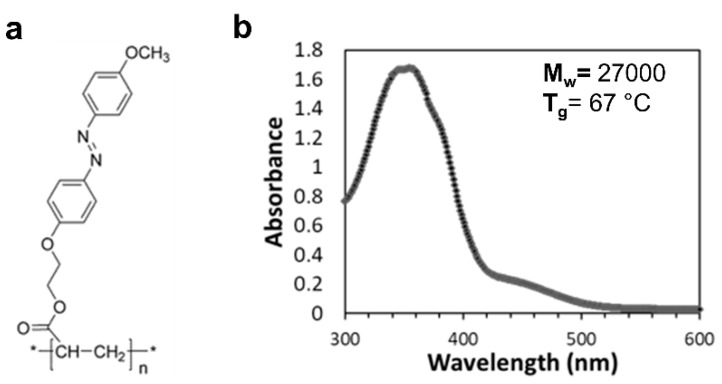
Chemical structure (**a**) and UV/visible absorption spectrum (**b**) of the azopolymer.

The photo-patternable polymeric substrate used in this work is an acrylic polymer with photoresponsive azobenzene moieties as side chains of the polymeric backbone. The sketch of the polymer chemical structure is shown in Figure 1a. Thermal analysis of the polymer performed by differential scanning calorimetry showed a glass transition temperature of 67° [12], while molecular weight measurements showed a molecular weight M_w_ = 27,000 and a dispersity index D_M_ = 1.28. Additional details about polymer synthesis and characterization can be found in references [12,14].

For the present experiments, transparent thin films (typical thickness 500 nm) were prepared by spin coating the solution of the polymer in 1,1,2,2-tetrachloroethane onto 170 µm thick microscope coverslips.

The UV/Vis absorption spectrum (acquired with PerkinElmer Lambda 900 spectrometer, PerkinElmer, Waltam, MA, USA) of the azopolymer films (Figure 1b) presents the typical azobenzene UV/Vis absorption features, which is characterized by two overlapped broad bands centered around 350 nm and 450 nm, originated respectively by the π-π* and n-π* transition of the azomolecules [11].

The photo-patterning abilities of the azopolymer used in the present work and the dependence of the light-induced surface reliefs on the intensity and polarization distribution of irradiated light field have been extensively investigated in several previous papers [12,14,16,30].

### 2.2. Photo-Induced Azopolymer Patterning and Topographical Characterization

The surface modulation of the azopolymer films was achieved by illuminating the samples with linearly polarized light of an Ar^+^ cw laser (λ = 488 nm). Two different type of samples were prepared: the first one (named “*single beam*” sample) is characterized by the spontaneous surface reliefs gratings (SSRGs) resulting from the light-induced self-structuration of the azopolymer film during the illumination with a single slightly focused laser beam [30,31,32,33]. To achieve this surface modulation, a collimated laser beam was first expanded to a diameter of 3 mm before being focused onto the sample surface through a cylindrical lens (focal length f = 75 cm). The resulting laser spot in the sample plane had an elliptic profile, with minimum width of 30 µm and length of about 2 mm. The light polarization direction was controlled by a half-wave plate and chosen to be parallel to the short axis of the ellipsis. The laser power was maintained constant at 0.870 mW for 1 h of illumination.

The second patterned substrates is constituted by the sinusoidal Surface Relief Gratings (SRGs), which are inscribed onto the surface of the sample “*grating*” by illuminating the azopolymer film with the periodic intensity pattern produced by the interference of two p-polarized laser beams [11]. This interference pattern was realized by recombining together in the film plane the two mutually coherent laser beams in which the primary laser beam was previously divided through a beam splitter. The angle between the k-vectors of the two interfering beams was properly adjusted in order to obtain a pitch of about 3.5 µm in the light interferogram and, consequently, in the SRGs induced onto the azopolymer film surface. The patterned sample area was about 4 × 4 mm^2^, while the power of the beams during the 1 h exposure time was P_1_ = 0.790 mW and P_2_=0.830 mW, respectively.

For both *single beam* and *grating* substrates, the morphology of the obtained light-induced superficial structures was characterized by Atomic Force Microscopy (AFM) (XE-100, Park Systems, Park Systems, Santa Clara, CA, USA). A simple pristine spin-coated azopolymer film (“*path-free*”) has been used as control substrate for comparison of cell behavior onto the unpatterned surface.

### 2.3. Cell Culture

Before cell seeding, scaffolds were sterilized in a 2% solution of penicillin/streptomycin for 5 h. Experiments were conducted with hMSCs (human mesenchymal stem cells) from LONZA (Merk, Switzerland), extracted from the adult patient’s bone marrow. These cells were grown in *Minimum Essential Medium Eagle—alpha modification* (α-MEM), supplemented with 10% fetal bovine serum (FBS), 2 mM L-glutamine, and 1% Penicillin/Streptomycin solution for 6 days on films with different patterns and with flat surfaces as control; then, they were seeded into 96-well plates with 1 × 10^4^ cells per scaffold.

### 2.4. Cell Viability Assay

Cell viability and proliferation were evaluated by using Alamar Blue® assay, which was based on a redox reaction that occurs in cells’ mitochondria. The main advantage of the Alamar Blue test was the ability to evaluate the response of live cells at different times, so monitoring cell proliferation over time on the same cell sample. Briefly, cell-scaffold constructs were removed from the culture plates at days 1, 2, and 6, washed with phosphate-buffered saline (PBS) (Sigma-Aldrich, Milan, Italy), and placed into 24-well culture plates. About 2 mL of Dulbecco’s modified Eagle’s medium without Phenol red (Hyclone) containing 10% (*v*/*v*) Alamar Blue (AbD Serotec Ltd., Milan, Italy) was added to each construct, and the samples were incubated for 4 h at 37 °C and 5% CO_2_. An aliquot of 200 µL of the solution was subsequently removed from the wells and transferred to a 96-well plate and analyzed by a spectrophotometer at wavelengths of 570 and 595 nm. The number of viable cells per scaffold was assessed by comparing the absorbance values at different cultures times with those of the calibration curve. The calibration curve was obtained by the correlation between a known cell number into the 24-well culture plates with the correspondent absorbance values.

### 2.5. Quantitative Bio-Imaging

The quantitative determination of key adherent cell culture characteristics, such as confluence, morphology, and cell density, is necessary for the evaluation of experimental outcomes and to provide a suitable basis for the establishment of cell culture protocols [26]. After 5 days in culture, cell morphology was investigated by Confocal Microscopy (Leica SP8,Wetzlar, Germany) supported by Cage incubator (Okolab, Naples, Italy) onto different patterns (*single beam*, *grating* and *path-free* as control). In this case, cells were formalin-fixed and stained with *phalloidin* (Alexa-Fluor 594, Invitrogen) and 4′,6-diamidino-2-phenylindole (DAPI) according to the manufacturer’s instructions. The elongation and orientation of cells were estimated from nuclei and actin filament cells by image elaboration via NIH ImageJ (freeware, ver.1.41). In this case, images were preliminary converted to grayscale, and the threshold was adjusted to highlight all of the cell nuclei to count. Then, background subtraction and watershed were applied to obtain a binary mask [27]. Lastly, the ‘Analyze Particle’ plugin was used to best fit the cell nuclei to elliptical shapes. Major and minor axes lengths of the closest-fitting ellipse were associated to cell nuclei lengths and widths, respectively. Elongation, defined as the extent of cell nuclei stretch, was calculated as the relative major to minor axis ratio or Aspect Ratio (AR), while orientation referred to the nuclei alignment and was calculated as slope of the tangent line to the ellipse respect to x-axis. Hence, the angle distribution of cell nuclei was elaborated.

Statistical analysis between each group was performed using the GraphPad Prism 5 program (GraphPad software, San Diego, CA, USA) and was determined using a Student’s *t*-test with significance levels set at 95% confidence interval. Lastly, cell sheet and actin filaments orientation were evaluated by using fast Fourier Transform (FFT) analysis (FIJI Directionality tool), and the orientation angle distribution was elaborated as reported elsewhere [28].

## 3. Results and Discussion

Micro and nanofabrication techniques based on photo-induced lithography currently represent an interesting technological approach to design micro and nanostructured surfaces with tailored topographic patterns. Despite their high spatial resolution, these techniques present some constrains related to high equipment costs, confinement of the structured area, and inability to further modify the fabricated topography under external stimuli (i.e., biological cues) [34]. In order to overcome some of these limitations, a great interest has recently arisen in the use of stimuli-responsive materials that are able to generate versatile and reconfigurable patterned surfaces, which are suitable as innovative models to investigate in vitro cell response to the environment of the extracellular matrix (ECM) [35]. Recently, azopolymers have been used to fabricate topographic patterns that are able to mimic the complex ECM architecture [36,37]. Herein, we aim at verifying the suitability of azopolymer-based patterns, which were fabricated via a very simple and cost-effective optical setup, to investigate the basic structural features—i.e., cell cytoskeleton, nuclei shape, and orientation—of hMSCs (human mesenchymal stem cells) in the interaction with a structured substrate due to the innate ability of cells to be highly sensitive to the underlying topographic signals. Biocompatibility and topographic effects on cell nuclei and morphology of actin filaments have been here investigated onto two structured azopolymer films having nanometric vertical features and diverse micrometric lateral patterns differing for the spatial extent of the structured area: parallel ripples, produced by the self-structuring phenomenon of the azopolymer films under irradiation with a slightly focused laser beam, and extended over a spatially confined line of the sample (the *single beam* sample) and periodic large-scale sinusoidal surface relief gratings (the *grating* sample). For negative control, also the interaction of hMSCs with the pristine unpatterned flat azopolymer film has been investigated. 

**Figure 2:**
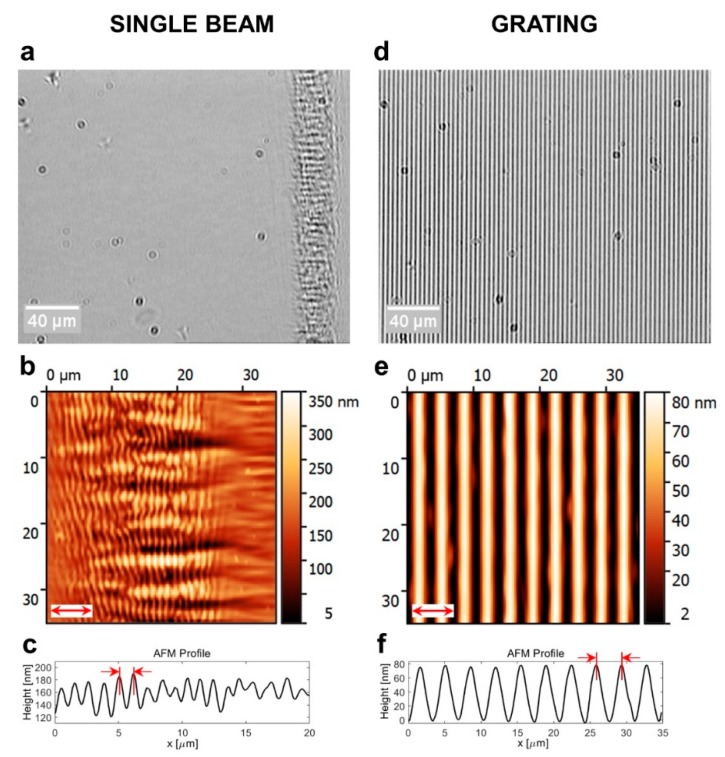
Morphological investigation of the light-structured azopolymer films: (**a**,**d**) optical images and (**b**,**e**) AFM images of *single beam* and *grating* samples, respectively. (**c**,**f**) Plot of AFM cross-section profiles used to evaluate the height and the periodicity of the structures.

The self-structured surface reliefs of the *single-beam* substrate—as shown in Figure 2a—have been induced in an elliptic region (~30 μm×2 mm) of the sample illuminated by means of a cylindrical lens (see Materials and Methods). The reliefs are characterized by a grating-like microstructuration showing a mean wavevector oriented in the direction of the laser polarization (indicated by the red arrow in Figure 2b. The height and the average periodicity of the grooves measured from the AFM profile—panel (c)—are $h_{1}\approx 100\;\mathrm{nm}$ and $\Lambda_{1}=1.14\;\mu m$, respectively.

Optical and AFM images—panels (d–e), respectively—of the *grating* substrate (see Materials and Methods) showed a periodic surface modulation, which was homogeneously extended in the whole illuminated area of about 4 mm in diameter. The SRGs had a wavevector oriented along the polarization direction (red arrow in Figure 2e) of the two laser beams used to produce the illuminating interferogram. The grating periodicity measured from AFM profile—as shown in panel (f)—was $\Lambda_{SRG}=3.5\;\mu m$, while the amplitude modulation was $h_{2}\approx 70\;\mathrm{nm}$.

**Figure 3:**
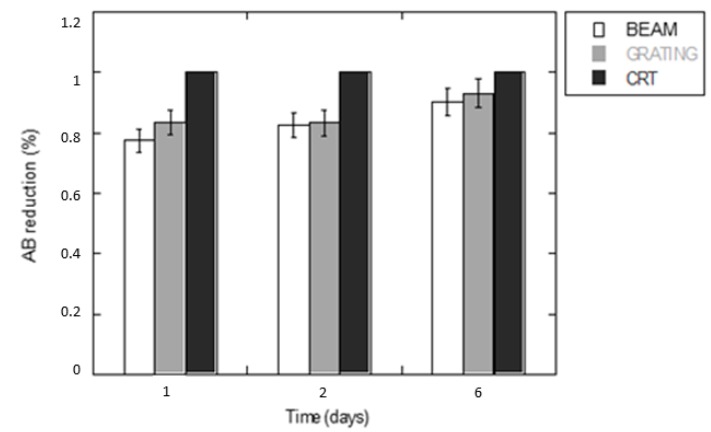
In vitro culture of human mesenchymal stem cells (hMSCs): viability at 1, 2, and 6 days (Alamar Blue test).

The spatial organization and the spatial extent of the grooves in the azopolymer substrates influences the cell interaction due to the capability of the cells to bind adhesive molecules to the substrate [38]. Before investigating the substrate-induced structural features of the cells, Alamar Blue® (AB) assay was used to evaluate the hMSCs’ viability and proliferation until 6 days as an index of the biocompatibility of the azopolymer films and eventual differences in the patterned areas of the samples. Results in terms of AB percentage reduction are shown in Figure 3. Despite a signal decay with respect to the polystyrene culture plate, no significant reduction of hMSCs between micropatterned substrates was observed over the time.

**Figure 4:**
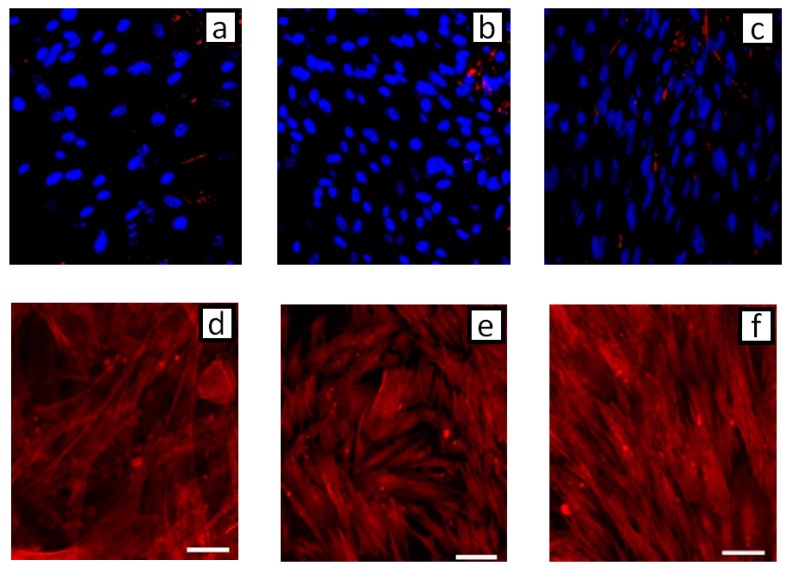
Fluorescently stained cellular nuclei (**a**–**c**) and actin filaments (**d**–**f**): confocal images of (**a**,**d**) unpatterned surface, (**b**,**e**) *single beam* and (**c**,**f**) *grating* samples.

**Figure 5:**
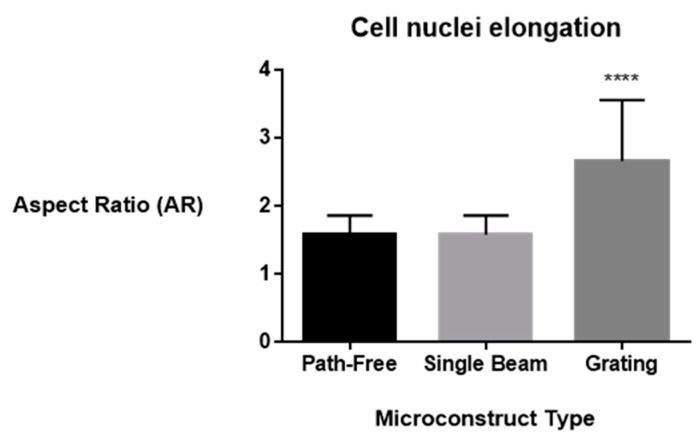
Aspect ratio of hMSCs nuclei on the unpatterned surface, *single beam* and *grating*. (**** *p* < 0.0001).

**Figure 6:**
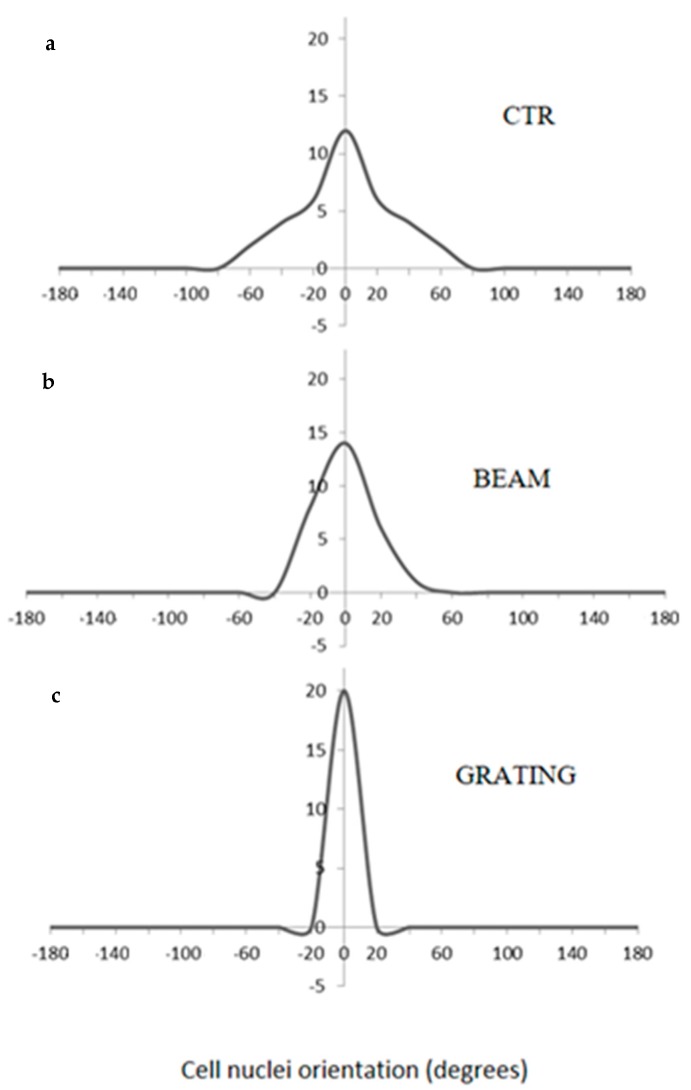
Cell nuclei orientation: (**a**) unpatterned surface, (**b**) *single beam* and (**c**) *grating*.

**Figure 7:**
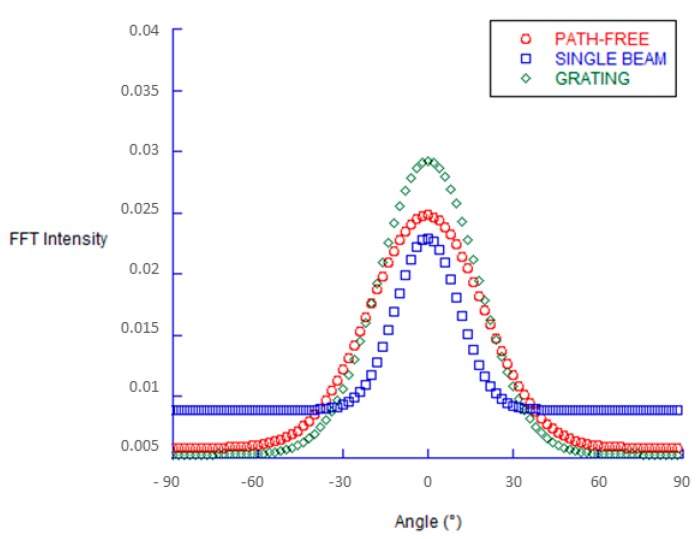
Fast Fourier Transform (FFT) analysis of actin filaments orientation and cytoskeletal organization.

According to the analysis reported in previous studies [39], the physiological state of cells was further investigated by confocal microscopy. Figure 4 showed the evidence of fluorescent stained cellular actin filaments and nuclei that confirm a good contact guidance mechanism with some differences due to the structural features of the underlying topographic patterns. In particular, hMSCs were mainly aligned parallel to the groove direction when seeded on patterned substrates (Figure 4b,c,e,f), with a larger effect produced by the periodic structuration of the *grating* sample compared to the *single beam*. On the contrary, the cells tended to show a random orientation onto the unpatterned surfaces (Figure 4a,d). Additionally, hMSCs cultured on the *grating* surface with periodic grooves (Figure 4c,f) showed an elongated cell nuclei morphology, which was not recorded in the case of the single-stripe samples (Figure 4b,e), thus suggesting a contribution of periodic and large-scale morphological signals at a micrometric scale on cell interaction. Accordingly, hMSCs nuclei on unpatterned surface (Figure 4a,d)—not including morphological signals—showed neither elongation nor orientation and were randomly spread. Microscale features initiate signals from the cell–matrix adhesions, which are basically transduced to the nucleus through the cytoskeletal network, from actin stress fibers to the intermediate filament network of the nucleus [28]. Hence, the structural changes of patterns at different size scales mainly induce a modification of hMSC nuclei morphologies. This was confirmed by different quantitative calculations on selected images. The AR value—which is used to measure nuclei elongation—was calculated in the case of the *grating* and *single-beam* azopolymer samples, showing AR values of 2.67 ± 0.90 and 1.58 ± 0.28, respectively, while an AR value equal to 1.59 ± 0.28 was obtained for the unpatterned surfaces (Figure 5). These results were totally in agreement with the theory of the contact guidance [38]. Indeed, cell alignment and elongation are mainly related to the probability that a cell might present a certain protrusion along a given direction [36]. Herein, cellular nuclei alignment angles were measured. Figure 6 compared the values of angles formed by the major axis of the fittest ellipse with the x-axis. The *single*-*beam* sample showed a narrower distribution compared to the unpatterned substrate. The distribution is further squeezed in the case of the *grating* sample, thus confirming an increase of cell alignment due to the periodic topographic stimulus of the sinusoidal SRGs. Meanwhile, the distribution of actin filaments orientation angles, being an important determinant of cellular shape and motility [38], was also calculated. FFT analysis of F-actin filaments performed onto selected confocal images, confirmed that newly formed filaments of the extracellular matrix were more elongated on the *grating* surface with respect to those on the *single-beam* and unpatterned films (Figure 7), corroborating the idea that periodic morphological signals strongly address cell morphology in vitro.

## 4. Conclusions

In this work, we have verified that photopatterned azopolymers films may be successfully used to address cell guidance mechanisms in vitro (i.e., cell aggregation, preferential elongation). Viability test confirmed that azopolymeric films with peculiar bioelectric/optical properties are biocompatible and support cell interaction. Moreover, qualitative and quantitative analyses indicated that cell nuclei and cytoskeletal actin filaments can be drastically influenced by the presence of grating-like superficial patterns. These results suggest the promising use of azopolymer films with controlled grating to design in vitro anisotropic models for the analysis of cell guidance mechanisms induced by topological signals in tissue engineering. For instance, studies have recently demonstrated that a biocompatible azopolymer can efficiently control the phenotype of neurons by influencing how cells respond to the nanometric grooves: i.e., by promoting a synaptic formation of multiple neurites or the elongation of single predominant neurites along preferential directions over time [40]. In the current scenario, complex topographies could be instructed on demand, under a controlled light stimulation, with arbitrary spatial distributions over a wide range of spatial and temporal scales [41]. In this perspective view, the route toward the design of bioinspired materials that are able to dynamically interact and/or instruct cells for innovative approaches in tumor diagnosis and in vivo cancer modeling can be traced.
